# Long Non-coding RNA *H19* Inhibits Adipocyte Differentiation of Bone Marrow Mesenchymal Stem Cells through Epigenetic Modulation of Histone Deacetylases

**DOI:** 10.1038/srep28897

**Published:** 2016-06-28

**Authors:** Yiping Huang, Yunfei Zheng, Chanyuan Jin, Xiaobei Li, Lingfei Jia, Weiran Li

**Affiliations:** 1 Department of Orthodontics, Peking University School and Hospital of Stomatology, Beijing 100081, China; 2 Department of Prosthodontics, Peking University School and Hospital of Stomatology, Beijing 100081, China; 3 Central Laboratory, Peking University School and Hospital of Stomatology, Beijing 100081, China; 4 National Engineering Laboratory for Digital and Material Technology of Stomatology, Beijing Key Laboratory of Digital Stomatology, Beijing 100081, China

## Abstract

Bone marrow mesenchymal stem cells (BMSCs) exhibit an increased propensity toward adipocyte differentiation accompanied by a reduction in osteogenesis in osteoporotic bone marrow. However, limited knowledge is available concerning the role of long non-coding RNAs (lncRNAs) in the differentiation of BMSCs into adipocytes. In this study, we demonstrated that lncRNA *H19* and *microRNA-675* (*miR-675*) derived from *H19* were significantly downregulated in BMSCs that were differentiating into adipocytes. Overexpression of *H19* and *miR-675* inhibited adipogenesis, while knockdown of their endogenous expression accelerated adipogenic differentiation. Mechanistically, we found that *miR-675* targeted the 3′ untranslated regions of the histone deacetylase (HDAC) 4–6 transcripts and resulted in deregulation of HDACs 4–6, essential molecules in adipogenesis. In turn, trichostatin A, an HDAC inhibitor, significantly reduced CCCTC-binding factor (CTCF) occupancy in the imprinting control region upstream of the *H19* gene locus and subsequently downregulated the expression of *H19*. These results show that the CTCF*/H19/miR-675/*HDAC regulatory pathway plays an important role in the commitment of BMSCs into adipocytes.

Marrow fat accumulation is characteristic of aging, estrogen deficiency, chemotherapy, glucocorticoid therapy, and radiotherapy^1^, and it is frequently associated with morbidity in osteoporosis^2^. Bone marrow fat cells and bone cells share a common progenitor, and they arise from bone marrow mesenchymal stem cells (BMSCs)^3^. An inverse relationship exists between marrow fat production and bone formation^4^. One possible cause of fat deposition is the aberrant commitment of BMSCs to the adipocyte lineage^5^,^6^. Thus, further understanding of the molecular mechanisms that initiate the differentiation of BMSCs into adipocytes may lead to the development of therapies that prevent excessive bone marrow adipogenesis and deficient bone formation.

Non-protein-coding RNAs (ncRNAs) have emerged as important epigenetic regulators in biological control and pathology^7^. A class of small ncRNAs has been identified as important regulators in the adipogenic differentiation of BMSCs. For example, *miR-188* regulates the age-related adipogenic differentiation of BMSCs and increases bone marrow adiposity^8^; overexpression of *miR-29a* protects against glucocorticoid-induced fatty marrow and bone loss by inhibiting histone deacetylase 4 (HDAC 4)^9^; and *miR-320* promotes the lineage-specific commitment of BMSCs to the adipocyte lineage by directly targeting RUNX2^10^. In contrast, the global expression patterns and functional contributions of long non-coding RNAs (lncRNAs), tentatively defined as ncRNAs >200 nucleotides (nt) in length^11^,^12^, in BMSC adipogenic differentiation are still largely unknown.

*H19* is a paternally-imprinted gene that does not encode a protein, but rather a 2.3-kb ncRNA^13^. It harbors an miRNA-containing hairpin and generates *miR-675* in a classic Drosha and Dicer splicing-dependent manner^14^. Recent studies have highlighted the role of *H19* in embryonic placental growth and cellular differentiation^15^,^16^,^17^. However, its functions in the adipogenic differentiation of BMSCs remain unclear. Here, we revealed that *H19* and its derivative *miR-675* inhibited adipogenesis in BMSCs by reducing the expression of class II HDACs 4, 5, and 6, essential molecules in adipogenesis. We also found that HDAC inhibition reduced *H19* expression by decreasing the CCCTC-binding factor (CTCF) occupancy on the imprinting control region (ICR) of *H19*. These results provide better understanding of lncRNA regulator involved in the commitment of BMSCs to adipocytes.

## Results

### *H19* and *miR-675* expression is downregulated during adipocyte differentiation

**Figure 1:**
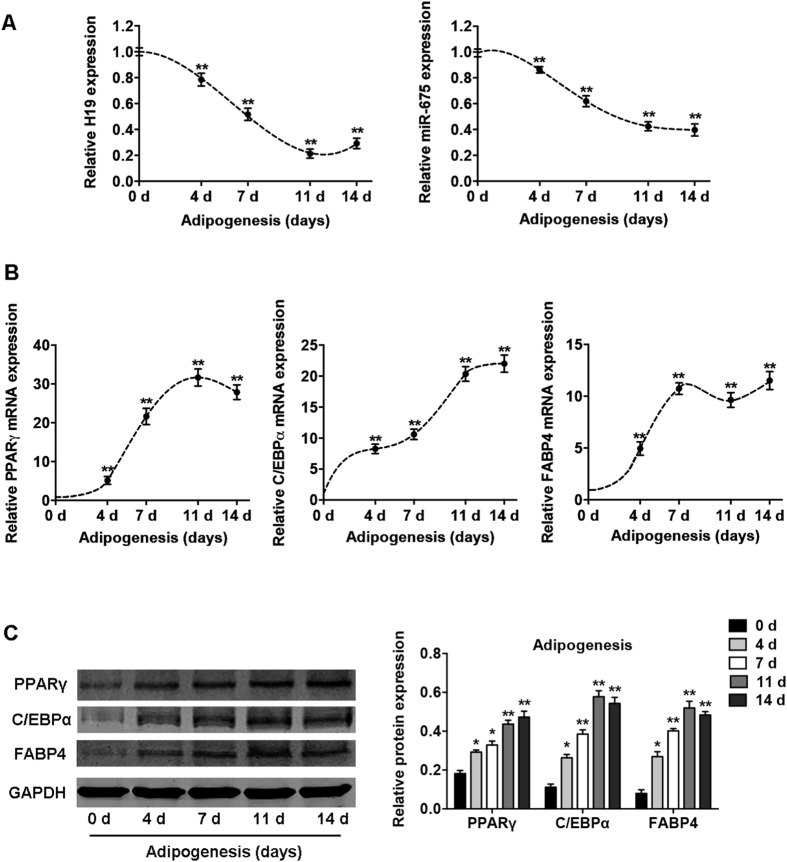
Expression profiles of lncRNA *H19* and *H19*-derived *miR-675* during adipogenic differentiation of BMSCs. (**A**) Left: relative expression of *H19* during differentiation as determined by qRT-PCR analysis. Right: relative *miR-675* transcript levels at the indicated time points during differentiation. (**B**) Relative mRNA expression levels of the adipogenic markers *PPARγ*, *C/EBPα*, and *FABP4* at the indicated time points, as in (**A**). RNA expression at the time points was normalized to that on day 0. (**C**) Western blot analysis (left) and quantification (right) of protein expression of PPARγ, C/EBPα, FABP4, and the internal control GAPDH at the indicated time points, as in (**A**). Results are presented as mean ± SD (*P < 0.05, **P < 0.01 compared to BMSCs without adipocyte induction).

The dynamic expression profiles of *H19* and *miR-675* were detected in BMSCs after their induction to the adipogenic lineage. Quantitative reverse-transcription (qRT-PCR) showed that *H19* expression was significantly reduced after adipogenic induction and decreased by 80% on day 11 (Fig. 1A). The expression of *miR-675*, derived from *H19*, showed a similar trend and decreased by 50% after adipocyte differentiation (Fig. 1A). The mRNA and protein expression of the genes associated with adipogenic differentiation, peroxisome proliferator-activated receptor-γ (*PPARγ*), CCAAT-enhancer-binding proteins-α (*C/EBPα*), and fatty-acid binding protein 4 (*FABP4*), was significantly upregulated after adipogenesis (Fig. 1B,C).

### Overexpression of *H19* and *miR-675* inhibits adipocyte differentiation

**Figure 2:**
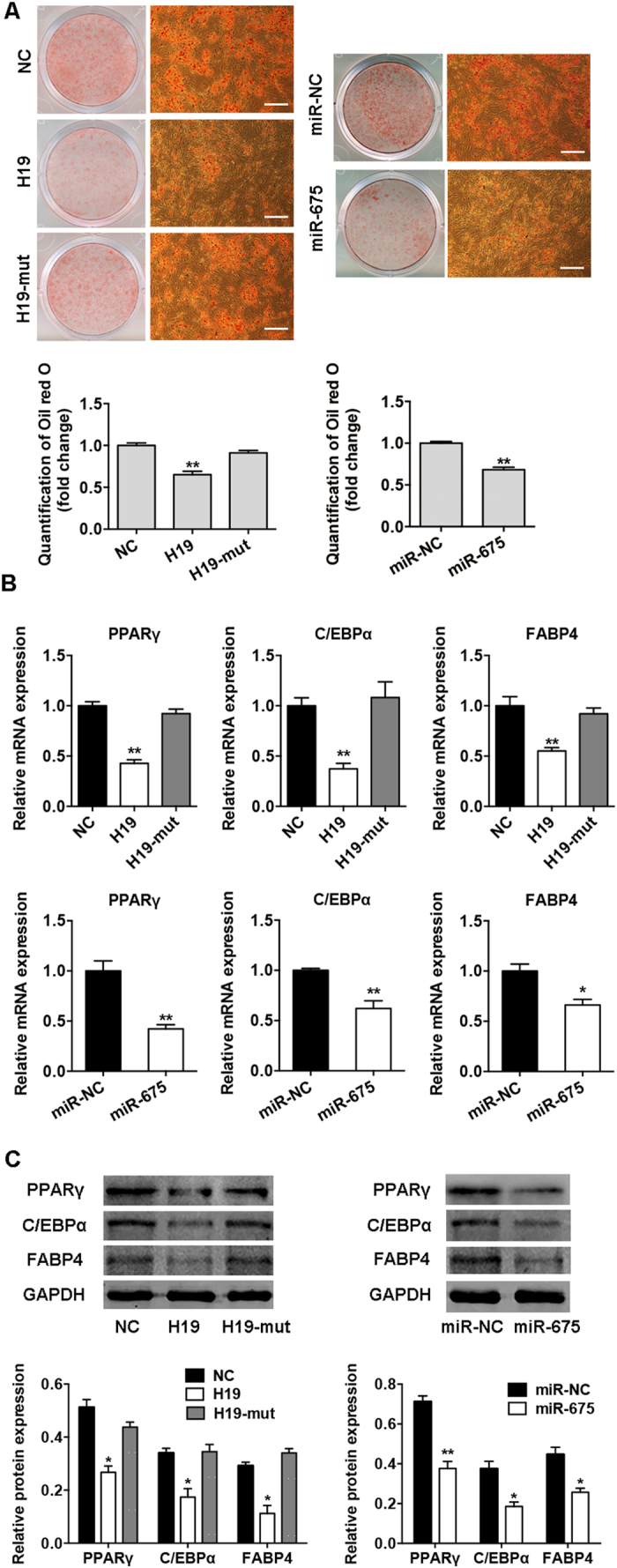
*H19* and *miR-675* inhibited adipogenic differentiation of BMSCs. (**A**) Images of Oil red O staining in BMSCs transfected with *H19*, *H19*-mut, *miR-675*, or their control vectors (NC, miR-NC) on day 10 of differentiation. Scale bar, 500 μm. Histograms show quantification of Oil red O staining by spectrophotometry (normalized to control groups). (**B**) Relative mRNA expression of the adipogenic factors *PPARγ*, *C/EBPα*, and *FABP4* measured by qRT-PCR on day 10 of adipogenic induction in BMSCs transfected with *H19*, *H19*-mut, *miR-675*, or their control vectors. (**C**) Western blot analysis of PPARγ, C/EBPα, FABP4, and GAPDH on day 10 of adipogenic induction in BMSCs transfected with *H19*, *H19*-mut, *miR-675*, or their control vectors. Histograms show quantification of the band intensities. Results are presented as mean ± SD (*P < 0.05, **P < 0.01).

LncRNA *H19* was located both in nucleus and cytoplasm, and it was enriched in the cytoplasmic fraction (Supplementary Figure S1A). To determine whether *H19* and *miR-675* directly affect adipocyte differentiation, lentiviruses were used to overexpress *H19* and *miR-675* in BMSCs. The efficiency of lentiviral transduction was >90% and the expression of *H19* and *miR-675* was significantly upregulated by >8-fold (Supplementary Figure S1B), as described previously^15^. After induction to the adipogenic lineage, *H19* and *miR-675* significantly inhibited intracellular lipid accumulation as indicated by Oil red O staining (Fig. 2A). The mRNA and protein expression of the adipocyte-specific factors *PPARγ*, *C/EBPα*, and *FABP4* was significantly reduced by the overexpression of *H19* and *miR-675* (Fig. 2B,C). To determine whether *H19* acts on adipogenesis *via miR-675*, we designed a mutant *H19* that carried mutation in the sequences of *miR-675*. The inhibition of adipocyte differentiation induced by *H19* was abrogated through mutation of *miR-675*, as indicated by Oil red O staining and the expression of adipocyte-specific genes (Fig. 2A–C).

### Knockdown of *H19* and *miR-675* promotes adipocyte differentiation

**Figure 3:**
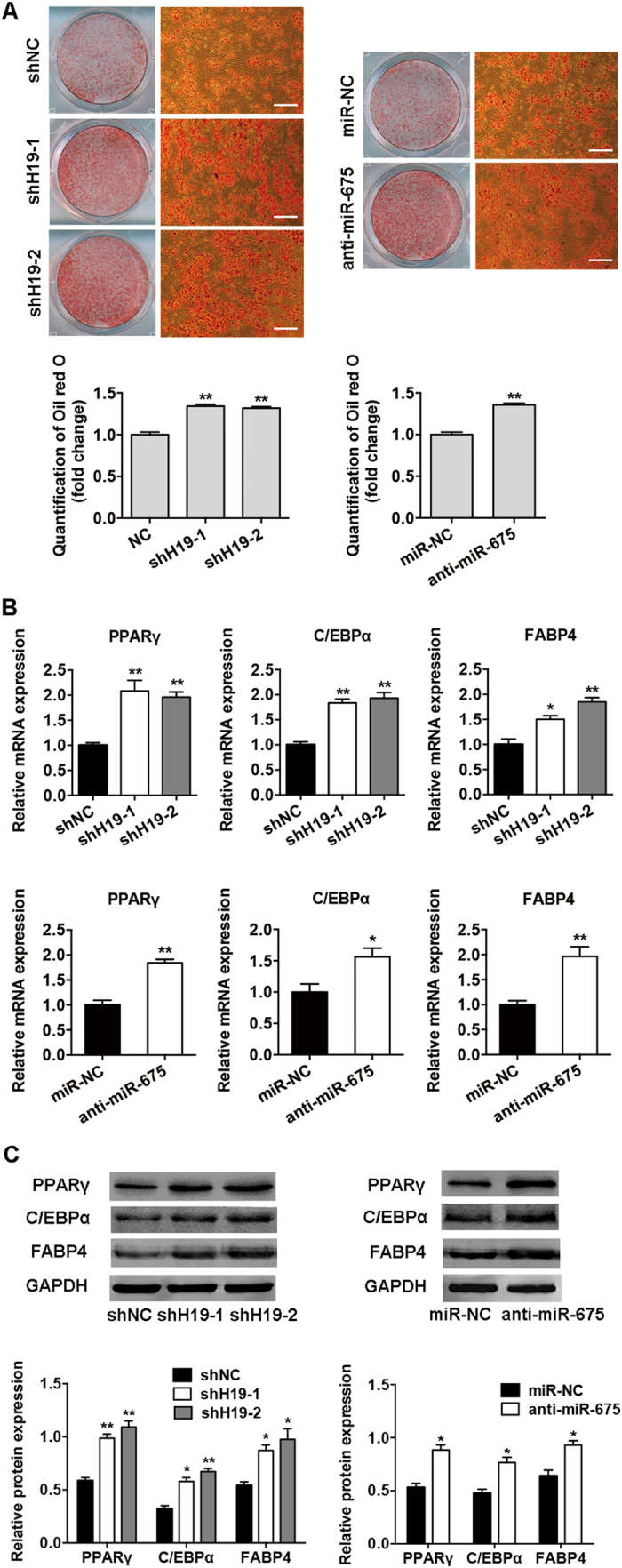
Knockdown of *H19* and *miR-675* promoted adipogenic differentiation of BMSCs. (**A**) Images of Oil red O staining in BMSCs transfected with sh*H19*, anti-*miR-675*, or their scrambled vectors (shNC, miR-NC) on day 10 of differentiation. Scale bar, 500 μm. Histograms show quantification of Oil red O staining by spectrophotometry (normalized to control groups). (**B**) Relative mRNA expression of the adipogenic genes *PPARγ*, *C/EBPα*, and *FABP4* on day 10 of differentiation in BMSCs transfected with sh*H19*, anti-*miR-675*, or their scrambled vectors. (**C**) Western blot analysis (upper) and quantification (down) of protein expression of PPARγ, C/EBPα, FABP4, and GAPDH on day 10 of differentiation in BMSCs transfected with sh*H19*, anti-*miR-675*, or their scrambled vectors. Data are presented as mean ± SD (*P < 0.05, **P < 0.01).

To further confirm the effects of *H19* and *miR-675* on adipocyte differentiation, we knocked down the endogenous *H19* and *miR-675* in BMSCs using lentivirus transfection. To control for potential off-target shRNA effects, we used two different shRNA sequences targeting *H19*. The expression of *H19* and *miR-675* was significantly reduced by ~70% (Supplementary Figure S1B), as described previously^15^. The inhibition of *H19* and *miR-675* promoted adipocyte formation as shown by Oil red O staining (Fig. 3A), and knockdown of *H19* and *miR-675* also significantly upregulated the mRNA and protein expression of the adipogenic marker genes *PPARγ*, *C/EBPα*, and *FABP4* (Fig. 3B,C).

### HDACs 4, 5, and 6 are directly targeted by *miR-675*

**Figure 4:**
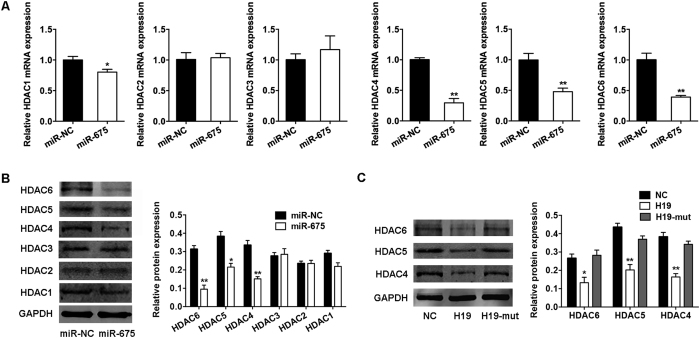
*H19* and *miR-675* downregulated the expression of HDACs 4, 5, and 6. (**A**) Quantification of mRNA expression of *HDACs 1–6* measured by qRT-PCR in BMSCs overexpressing *miR-675* relative to the miRNA control group (miR-NC). (**B**) Western blot analysis of HDACs 1–6 and GAPDH in BMSCs overexpressing *miR-675* or miR-NC. Histograms show quantification of the band intensities. (**C**) Western blot analysis of HDACs 4–6 and GAPDH in BMSCs overexpressing *H19*, *H19*-mut, and the control vector (NC). Histograms show quantification of the band intensities. Results are presented as mean ± SD (*P < 0.05, **P < 0.01).

HDACs respond to signals that regulate a broad and complex array of physiological processes, including adipocyte differentiation and metabolism^18^. Our previous study showed that *H19* and *miR-675* reduce the expression of HDACs 4 and 5^15^, but the mechanism remains unclear. So, we further assessed the expression of HDACs 1–6 in BMSCs overexpressing *miR-675*. Compared with the negative control, *miR-675* substantially reduced the mRNA and protein levels of class II HDACs 4, 5, and 6, and slightly inhibited the expression of HDAC 1 (by ~20%), whereas no effect on the expression of HDACs 2 and 3 was found (Fig. 4A,B). Consistently, the ectopic overexpression of *H19* downregulated the expression of HDACs 4, 5, and 6 in BMSCs, and the repression was relieved through mutation of *miR-675* sequences (Fig. 4C).

**Figure 5:**
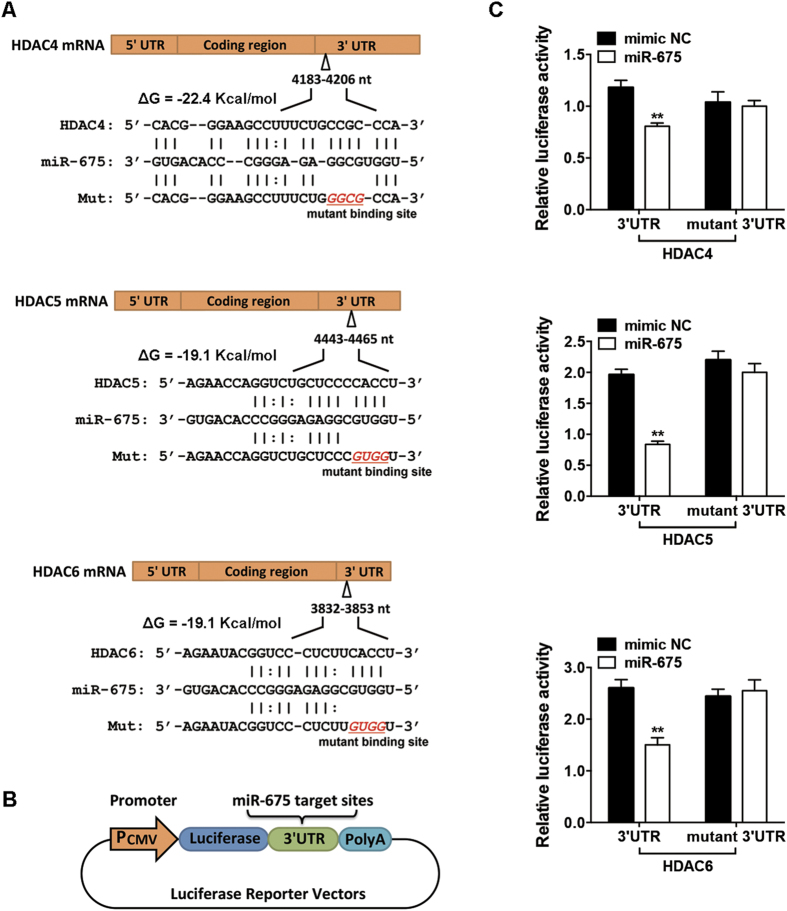
*miR-675* directly targeted the 3′ untranslated regions (UTRs) of HDAC 4, 5, and 6 transcriptions. (**A**) Schematic diagrams of the wild-type and mutant binding sites of *miR-675* located in the 3′UTRs of HDAC 4, 5, and 6 transcriptions. (**B**) Schematic showing the constructed luciferase reporter system containing the binding sites of *miR-675*. (**C**) Luciferase activity of 293T cells co-transfected with 100 nM *miR-675* mimic or miRNA mimic control (mimic NC) and the luciferase constructs carrying the 3′UTR of HDAC 4, 5, or 6. Results are shown as mean ± SD (**P < 0.01).

We then performed bioinformatic predictions of miRNA targets using RNA22 software^19^. The transcripts of HDACs 4–6 were found to contain several putative *miR-675*-binding sites (Supplementary Tables S1–S3). Among these, *miR-675* possessed the maximum likelihood of binding to the 3′ untranslated region (UTR) of HDAC 4 (4183–4206 nt) (ΔG = −22.4 kcal/mol), the 3′UTR of HDAC 5 (4443–4465 nt) (ΔG = −19.1 kcal/mol), and the 3′UTR of HDAC 6 (3832–3853 nt) (ΔG = −19.1 kcal/mol) (Fig. 5A). Thus, we chose these target sites and separately ligated them to a luciferase reporter (Fig. 5B) to determine whether *miR-675* directly targets these sites. The ectopic overexpression of *miR-675* significantly inhibited luciferase activity in the HDAC 4, 5, and 6 constructs (Fig. 5C). Mutation of the *miR-675*-binding site abolished the inhibitory effect of *miR-675* on the HDAC 4, 5, and 6 reporter activity (Fig. 5C).

### Knockdown of HDACs 4, 5, and 6 inhibits adipocyte differentiation

**Figure 6:**
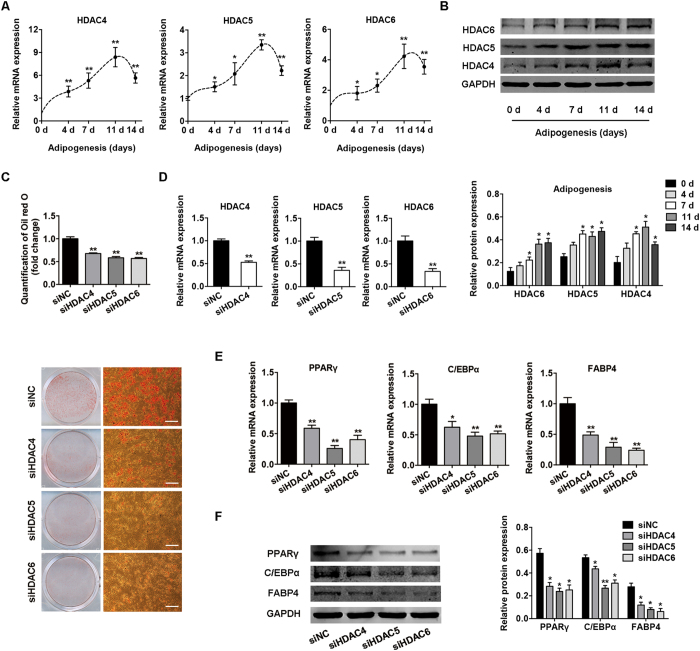
Knockdown of HDACs 4–6 by RNA interference suppressed the adipogenic differentiation of BMSCs. (**A**) Relative mRNA expression levels of *HDACs 4–6* at the indicated time points during adipocyte differentiation. (**B**) Western blot analysis of HDACs 4–6 and GAPDH at the indicated time points during adipogenesis. Histograms show quantification of the band intensities. RNA and protein expression at each time point was compared to the group without adipocyte induction. (**C**) Images of Oil red O staining in BMSCs transfected with specific siRNAs on day 7 of adipogenic differentiation. Scale bar, 500 μm. Histograms show quantification of Oil red O staining normalized to the siNC group. (**D**) Quantification of mRNA expression of *HDACs 4*–*6* measured by qRT-PCR after transfection with siHDAC4-6 or siNC. (**E**) Relative mRNA expression of the adipocyte-specific genes *PPARγ*, *C/EBPα*, and *FABP4* measured by qRT-PCR on day 7 of adipogenic induction in BMSCs transfected with specific siRNAs. (**F**) Western blot analysis of PPARγ, C/EBPα, FABP4, and GAPDH on day 7 of adipogenic induction in BMSCs transfected with specific siRNAs. Histograms show quantification of the band intensities. Data are shown as mean ± SD (*P < 0.05, **P < 0.01).

To determine the role of HDACs 4, 5, and 6 in adipogenesis, we first measured their expression patterns during adipocyte differentiation. The expression of HDACs 4–6 was gradually upregulated with the highest expression on day 11 (Fig. 6A,B), which was inversely correlated with *H19* expression during adipogenesis. We then used specific small-interfering RNAs (siRNAs) to suppress their endogenous expression. The specific siRNAs were transfected into BMSCs in growth medium, which was changed to adipogenic-differentiation medium on day 1. On day 3, the siRNAs were transfected again, and the cells were harvested on day 7. Successful knockdown of HDACs 4, 5, and 6 (Fig. 6D) inhibited the adipocyte differentiation of BMSCs as indicated by Oil red O staining (Fig. 6C), and the mRNA and protein expression of adipogenic genes *PPARγ*, *C/EBPα*, and *FABP4* was also significantly downregulated (Fig. 6E,F).

### Trichostatin A (TSA) reduces CTCF occupancy in the *H19* imprinting control region (ICR) and downregulates *H19* expression

**Figure 7:**
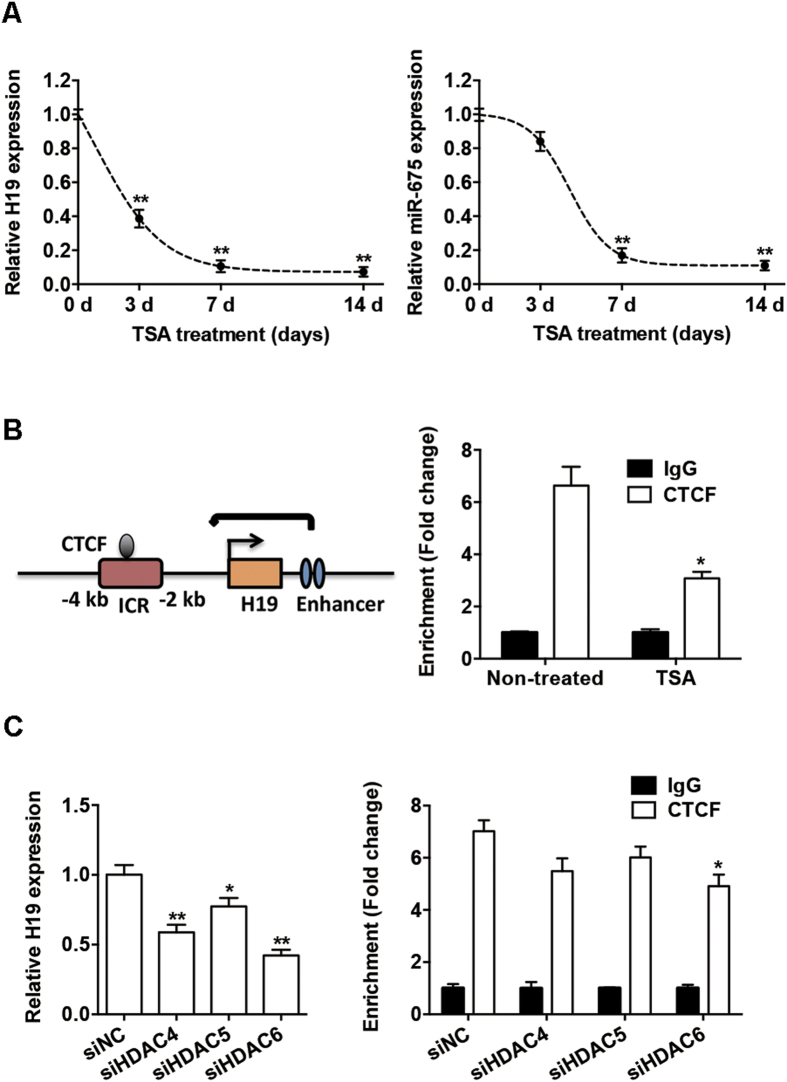
HDAC inhibition reduced the binding of CTCF in the *H19* imprinting control region (ICR). (**A**) Left: relative expression of *H19*, as determined by qRT-PCR analysis, in BMSCs with or without TSA (400 nM) for 3, 7, and 14 days. Right: relative *miR-675* expression in BMSCs with or without TSA (400 nM) for 3, 7, and 14 days. (**B**) Left: schematic of the *H19* gene locus. Right: ChIP assay of CTCF occupancy in the *H19* ICR. Soluble chromatin from BMSCs with or without TSA was immunoprecipitated with CTCF or IgG antibodies, and the immunoprecipitated DNA was analyzed by qRT-PCR. (**C**) Left: relative expression of *H19*, as determined by qRT-PCR analysis, in BMSCs transfected with siHDAC4-6 or siNC. Right: ChIP assay of CTCF occupancy in the *H19* ICR from BMSCs transfected with siHDAC4-6 or siNC. Data are shown as mean ± SD (*P < 0.05, **P < 0.01, compared with non-treated or siNC group).

*H19* is an imprinted gene, and its expression is regulated by chromatin structure and epigenetic mechanisms^20^,^21^. Thus, we sought to determine the epigenetic effect of HDACs on the expression of *H19* and *miR-675*. We treated BMSCs with TSA (400 nM), an HDAC inhibitor, for 3, 7, and 14 days. This treatment significantly suppressed *H19* expression in a time-dependent manner. The expression of *H19* was significantly reduced after 3 days, while further treatment caused further reduction and *H19* expression was suppressed to a low level after 7 days (Fig. 7A). The expression of *miR-675* was also downregulated after TSA treatment, and displayed a pattern similar to *H19* expression (Fig. 7A).

*H19* expression is controlled by CTCF binding to the ICR upstream of the *H19* gene. The CTCF protein promotes enhancer function at the *H19* promoter (Fig. 7B)^22^,^23^. To determine whether TSA treatment leads to changes in the CTCF-binding status at the *H19* ICR, we analyzed the levels of CTCF occupancy in this region using chromatin immunoprecipitation (ChIP). Indeed, endogenous CTCF directly interacted with the *H19* ICR in BMSCs. And following TSA treatment, the occupancy of CTCF protein in the *H19* ICR was reduced (Fig. 7B).

TSA inhibits the enzymatic HDAC activity without significant change of their expression (Supplementary Figure S2). To confirm the role of HDACs in *H19* transcription, we applied siRNA gene knockdown studies and found that knockdown of HDACs 4–6 resulted in a phenotype similar to that observed after TSA treatment. *H19* expression was reduced by HDAC 4–6 knockdown, and CTCF occupancy in *H19* ICR was also moderately reduced with significant change in HDAC6 knockdown group (Fig. 7C).

## Discussion

In this study, we demonstrated that lncRNA *H19* and *H19*-derived *miR-675* were significantly downregulated in BMSCs that were differentiating into adipocytes. Overexpression of *H19* and *miR-675* in BMSCs inhibited adipogenesis, while knockdown of their expression accelerated adipogenic differentiation. These phenomena indicate that *H19* plays key roles in the process of BMSC adipogenesis. A few lncRNAs, such as *ADINR*^24^, *PU.1-as*^25^, and *NEAT1*^26^, have been found to directly participate in the genetic control of adipogenic differentiation in adipose-derived stem cells. However, the stem cells from different tissues differ in their metabolic activity and ability to differentiate^27^,^28^. The roles of lncRNAs in BMSC adipogenesis are still largely unknown. It has been reported that when the balance between adipogenesis and osteogenesis is disturbed and BMSCs tend to differentiate into adipocytes rather than osteoblasts, marrow fat progressively accumulates and bone loss occurs^4^,^5^,^6^. Several studies have identified regulators of the switch between osteogenesis and adipogenesis in BMSCs, such as *miR-188*^8^, *Maf *29, *Ezh2*, and *Kdm6a*^30^. Here, we demonstrated that *H19* plays inhibitory roles in adipogenesis, while our previous study showed that *H19* promotes osteoblast differentiation^15^. Thus, *H19* seems to function in the switch of osteoblast and adipocyte differentiation of BMSCs. In a preclinical mouse model, BMSC-specific inhibition of *miR-188* by intra-bone marrow injection of aptamer-antagomiR-188 increases bone formation and decreases bone marrow fat accumulation^8^. Our studies indicating a role of lncRNA *H19* in the shift from adipogenesis to osteogenesis in BMSCs may also provides a potential target for treating fatty marrow and bone loss.

LncRNAs exert their functions *via* diverse mechanisms, including co-transcriptional regulation, modulation of gene expression, scaffolding of nuclear or cytoplasmic complexes, and pairing with other RNAs^11^. Another means by which lncRNAs acquire functionality is by acting as precursors of miRNAs^31^ or as sinks for pools of active miRNAs^32^ to regulate transcripts targeted by that set of miRNAs. Several previous studies have shown that *miR-675* confers functionality on *H19*^14^,^16^,^17^,^33^. Our results addressing the inhibitory effect of *miR-675* on adipogenic differentiation and lost of function in *H19*-mut group suggest that *miR-675* is at least partially responsible for the inhibition of adipogenesis induced by *H19*. However, as lncRNAs act *via* diverse mechanisms, *H19* may interact with other molecules involved in the complex biology of adipogenic differentiation. In this study, we used PPARγ, FABP4, and C/EBPα as adipocyte markers, all of which were significantly upregulated during adipocyte differentiation. However, a previous study has showed that FABP4 is induced by PPARγ but negatively regulates PPARγ activity in macrophages and adipocytes^34^. There is no consensus on the interrelationship between FABP4 and PPARγ, and our results were consistent with some previous studies concerning adipocyte differentiation^24^,^35^,^36^,^37^. The discrepancy of FABP4 expression in previous studies may be attributable to the different cells and tissues.

We found that *miR-675* bound directly to the 3′UTRs of class II HDACs 4–6 and downregulated their mRNA and protein levels, while minor effects were found on the expression of class I HDACs 1, 2, and 3. The expression of HDACs 4–6 was inversely correlated with *H19* and *miR-675* expression during adipocyte differentiation. The downregulation of class II HDACs 4, 5, and 6 inhibited the adipogenic differentiation of BMSCs. Consistent with our results, previous studies have demonstrated the essential role of class II HDACs in adipocyte differentiation. Growing evidence shows that class I is directly involved in regulation of cell growth and apoptosis, whereas class II members regulate differentiation processes^38^. Treatment of mesenchymal stem cells with pan-HDAC inhibitors, class II-specific inhibitors, or specific siRNAs targeting HDACs, attenuates adipogenesis and reduces the expression of adipocyte markers following the induction of differentiation^38^,^39^,^40^,^41^,^42^. *H19* and *miR-675* inhibited the adipogenic differentiation of BMSCs at least partially through the downregulation of HDACs.

HDAC inhibition reduced CTCF enrichment in the *H19* ICR and downregulated *H19* expression. *H19* has been reported to be regulated by chromatin structure and epigenetic mechanisms, including DNA methylation, CTCF insulator, and enhancer activity^20^,^21^. Correct positioning of nucleosomes within the ICR is required for CTCF stably binding, which promotes enhancer function at the H19 promoter^22^,^23^. TSA treatment reduced the occupancy of the CTCF protein in the *H19* ICR and abolished the boundary activity of the ICR, thereby downregulating *H19* expression. There is evidence that regional changes in acetylation within the promoter of *H19* occur after TSA treatment, and *H19* expression is significantly reduced^43^. And there is also evidence that HDACs from nuclear extracts are bound by the CTCF zinc-finger domain, and CTCF may function by associating with the HDAC complex^44^. From these results, it is not clear whether the reduced CTCF occupancy is causally related to the histone acetylation status of H19 promoter, but it implies the role of HDACs in this lncRNA transcription. Also, knockdown of HDACs 4–6 resulted in the similar but weaker phenotype compared to TSA treatment, indicating that other histone or non-histone acetylation may be involved. The precise molecular mechanism that underlies the regulation of specific histone variations needs further investigation.

**Figure 8:**
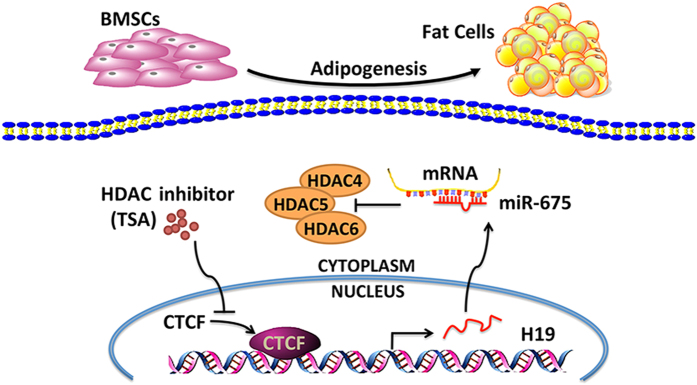
Schematic of pathways involved in the inhibition of BMSC adipogenic differentiation by *H19* and *miR-675*. *H19*-derived *miR-675* directly targeted HDACs 4, 5, and 6. TSA, an HDAC inhibitor, reduced the levels of CTCF occupancy in the *H19* imprinting control region and reduced *H19* expression.

In conclusion, *H19* and *H19*-derived *miR-675* inhibits the adipocyte differentiation of BMSCs through the epigenetic modulation of HDACs; *miR-675* directly targets HDACs 4, 5, and 6; and the inhibition of HDACs reduces the levels of CTCF occupancy in the *H19* ICR and reduces *H19* expression (Fig. 8). Further research may elucidate whether *H19* and *miR-675* modulate the shift of cell lineage commitment of BMSCs *in vivo* and provide a potential therapeutic target for bone marrow adiposity.

## Methods

### Cell cultures and adipocyte differentiation

Primary BMSC lines from three donors were obtained from ScienCell (San Diego, CA, USA) and cultured at sub-confluent density in growth medium consisting of α-minimum essential medium supplemented with 10% fetal bovine serum and 1% antibiotics. All cell-based *in vitro* experiments were repeated in triplicate. For the adipocyte differentiation experiment, cells were allowed to become confluent for 1 day, and then cultured in standard growth medium supplemented with 50 nM insulin (Sigma-Aldrich, Saint Louis, MO, USA), 100 nM dexamethasone (Sigma-Aldrich), 0.5 mM 3-isobutyl-1-methylxanthine (Sigma-Aldrich), and 200 μM indomethacin (Sigma-Aldrich). The adipogenic medium was changed every 2 days, and cells were harvested at the indicated times. The 293T cells were obtained from the American Type Culture Collection (Manassas, VA, USA) and cultured in Dulbecco’s modified Eagle’s medium with 10% fetal bovine serum and 1% antibiotics.

### Lentivirus infection

Recombinant lentiviruses harboring full-length *H19*, mutant *H19* (*H19*-mut), or *H19*-targeting sequences (sh*H19*-1 and sh*H19*-2) were constructed as described previously^15^. Site-directed mutagenesis of the *H19* sequences was performed using the Site-Directed Mutagenesis Kit (SBS Genetech, Beijing, China). It carried mutation in the sequences of *miR-675*, as follows: TGG TGC GGA GAG GGC CCA CAG TG was changed to TCC ACG CGA GAG GGC CCA CAG TG. A vacant lentiviral vector (NC) and a scrambled non-targeting vector (shNC) were used as control group. Recombinant lentiviruses harboring *miR-675* or *miR-675* inhibitor sequences (anti-*miR-675*) and the control vector (miR-NC) were obtained from Integrated Biotech Solutions Co. (Shanghai, China). Transfection of the BMSCs was performed by exposing them to dilutions of the viral supernatant in the presence of polybrene (5 μg/ml) for 24 h.

### Oil red O staining

The cells were washed with phosphate-buffered saline (PBS) and fixed in 10% formalin for 30 min. The cells were then rinsed with 60% isopropanol. Oil red O (0.3%, Sigma-Aldrich) was added and incubated for 10 min with gentle agitation. After staining, the cells were washed with distilled water to remove unbound dye, visualized by light microscopy, and photographed. For quantitative assessment, the Oil red O was eluted by 100% isopropanol and quantified by spectrophotometric absorbance at 520 nm against a blank (100% isopropanol).

### RNA oligoribonucleotides and chemicals

A chemically-modified double-stranded *miR-675* mimic and the corresponding miRNA mimic control (mimic NC) were obtained from RiboBio Co. (Guangzhou, China). siRNAs targeting HDAC 4, 5, and 6 transcripts (siHDAC4–6) and the corresponding siRNA control (siNC) were from Integrated Biotech Solutions Co. The sequences are listed in Supplementary Table S4. TSA (Sigma-Aldrich) was dissolved in dimethyl sulfoxide.

### Transient transfection

Cells at 70–80% confluence were transfected with plasmids, miRNA mimics, or siRNAs using Lipofectamine 2000 (Invitrogen, Carlsbad, CA, USA) according to the manufacturer’s instructions as described previously^45^.

### Reporter vectors

Predicted *miR-675* target genes and their target binding sites were investigated using RNA22 software^19^. The reporter vectors were constructed by Integrated Biotech Solutions Co. Briefly, the 3′UTRs of HDAC 4, 5, and 6 mRNA, containing the predicted *miR-675* binding sites, were synthesized and cloned into a modified version of pcDNA3.1(+) containing a firefly luciferase reporter gene (a gift from Professor Brigid L.M. Hogan, Duke University, Durham, NC, USA)^46^, at a position downstream of the luciferase reporter gene as described previously^45^. Site-directed mutagenesis of selected putative seeding-sequence regions was performed using the Site-Directed Mutagenesis Kit from SBS Genetech. All constructs were confirmed by DNA sequencing.

### Dual luciferase reporter assay

Luciferase assays were performed as described previously^45^. Briefly, 293T cells grown in 48-well plates were transfected with 100 nM *miR-675* mimic or control, 40 ng luciferase reporter, and 4 ng pRL-TK, a plasmid expressing *Renilla* luciferase (Promega, Madison, WI, USA) using Lipofectamine 2000 (Invitrogen). The *Renilla* and firefly luciferase activities were measured 24 h after transfection using the Dual Luciferase Reporter Assay System (Promega). All luciferase values were normalized to those of *Renilla* luciferase and expressed as fold-induction relative to the basal activity.

### RNA isolation and qRT-PCR

Total RNA was extracted using TRIzol reagent (Invitrogen) according to the manufacturer’s instructions and then reverse-transcribed into cDNA using the cDNA Reverse Transcription Kit from Applied Biosystems (Foster City, CA, USA). qRT-PCR was conducted using SYBR Green Master Mix on the ABI Prism 7500 real-time PCR System (Applied Biosystems) as described^45^. The following thermal settings were used: 95 °C for 10 min followed by 40 cycles of 95 °C for 15 s and 60 °C for 1 min. The primers used for *H19*, *miR-675*, *HDACs 1–6*, *PPARγ*, *C/EBPα*, *FABP4*, *U6* (internal control for miRNAs), and glyceraldehyde 3-phosphate dehydrogenase (*GAPDH*, internal control for mRNAs and lncRNAs) are listed in Supplementary Table S4. The data were analyzed using the 2^−ΔΔCt^ relative expression method as described previously^45^.

### Cell fractionation

Cytoplasmic and nuclear RNAs were fractionated using a Nuclei Isolation Kit (KeyGEN, Nanjing, China). Briefly, cells were harvested in lysis buffer, treated with Reagent A, incubated on ice for 15 min, followed by centrifugation at 4 °C. The pellet was then resuspended in lysis buffer followed by centrifugation. The supernatant was transferred to a new tube as the cytoplasmic fraction; the pellet was resuspended in Medium Buffer A and then added to a new tube with Medium Buffer B, followed by centrifugation at 4 °C. The supernatant was saved as the cytoplasmic fraction. The pellet was used as the nuclear fraction. RNA was extracted from both fractions using TRIzol.

### Western blot analysis

Western blot analysis was performed as described previously^45^. Briefly, cells were harvested, washed with PBS, and lysed in RIPA buffer. Proteins were separated by 10% sodium dodecyl sulfate–polyacrylamide gel electrophoresis and transferred to nitrocellulose membranes. Primary antibodies against PPARγ (Cell Signaling Technology, Beverly, MA, USA), HDACs 1–6 (Cell Signaling Technology), C/EBPα (HuaxingBio Science, Beijing, China), FABP4 (HuaxingBio Science), and GAPDH (Abcam, Cambridge, UK) were diluted 1:1,000. The intensities of the bands obtained by Western blot analysis were quantified using ImageJ software (http://rsb.info.nih.gov/ij/). The background was subtracted, and the signal of each target band was normalized to that of the GAPDH band.

### ChIP assay

ChIP assays were performed using the EZ-Magna ChIP assay kit (Merck Millipore, Darmstadt, Germany) according to the manufacturer’s instructions. Briefly, cells were washed with PBS and cross-linked with 1% formaldehyde for 10 min. Chromatin was sonicated on ice to generate chromatin fragments of 500–2000 bp. Then the DNA-protein complexes were isolated using antibodies against CTCF (Cell Signaling Technology) or isotype IgG (Cell Signaling Technology). The protein/DNA complexes were then eluted and reverse cross-linked. Input control DNA or immunoprecipitated DNA was quantified by qRT-PCR, using SimpleChIP Human *H19*/*IGF2* ICR Primers (Cell Signaling Technology). Relative enrichment was calculated as the amount of amplified DNA normalized to the input and relative to values obtained after IgG immunoprecipitation.

### Statistical analysis

Statistical analyses were performed using SPSS version 16.0 (SPSS, Chicago, IL, USA). All data are expressed as mean ± standard deviation (SD). Differences between groups were analyzed using Student’s *t*-test. In cases of multiple-group testing, one-way analysis of variance was conducted. A two-tailed value of *P* < 0.05 was considered statistically significant.

## Additional Information

**How to cite this article**: Huang, Y. *et al*. Long Non-coding RNA *H19* Inhibits Adipocyte Differentiation of Bone Marrow Mesenchymal Stem Cells through Epigenetic Modulation of Histone Deacetylases. *Sci. Rep.* **6**, 28897; doi: 10.1038/srep28897 (2016).

## Supplementary Material

Supplementary Information
