# Supplementary material for: Long Non-coding RNA H19 Inhibits Adipocyte Differentiation of Bone Marrow Mesenchymal Stem Cells through Epigenetic Modulation of Histone Deacetylases
Source: Sci Rep. 2016 Jun 28;6:28897. doi: 10.1038/srep28897 (PMC4924093; doi:10.1038/srep28897)
Supplement: Supplementary Information [file srep28897-s1.pdf]

Long Non-coding RNA *H19* Inhibits Adipocyte Differentiation of Bone Marrow Mesenchymal Stem Cells through Epigenetic Modulation of Histone Deacetylases

Yiping Huang[1], Yunfei Zheng[1], Chanyuan Jin[2], Xiaobei Li[1], Lingfei Jia[1, 3*,] Weiran Li[1, 4*]

[1]Department of Orthodontics, Peking University School and Hospital of Stomatology, Beijing 100081, China.

[2]Department of Prosthodontics, Peking University School and Hospital of Stomatology, Beijing 100081, China.

[3]Central Laboratory, Peking University School and Hospital of Stomatology, Beijing 100081, China.

[4]National Engineering Laboratory for Digital and Material Technology of Stomatology, Beijing Key Laboratory of Digital Stomatology, Beijing 100081, China

Supplementary Information

Supplementary Figure S1. *H19* and *miR-675* expression in BMSCs transfected with lentiviruses. (A) Percentage of nuclear and cytoplasmic RNA levels of *H19*, *MALAT1*, and *GAPDH* measured by qRT-PCR after subcellular fractionation in BMSCs. (B) Left: relative expression of *H19*, as determined by qRT-PCR analysis, in BMSCs transfected with *H19* and the vacant vector (NC). Middle: relative expression of *H19* in BMSCs transfected with sh*H19* and the non-targeting vector (shNC). Right: relative expression of *miR-675* in BMSCs transfected with *miR-675*, anti-*miR-675*, or the scrambled vector (miR-NC). Data are shown as mean ± SD (***P* <0.01).

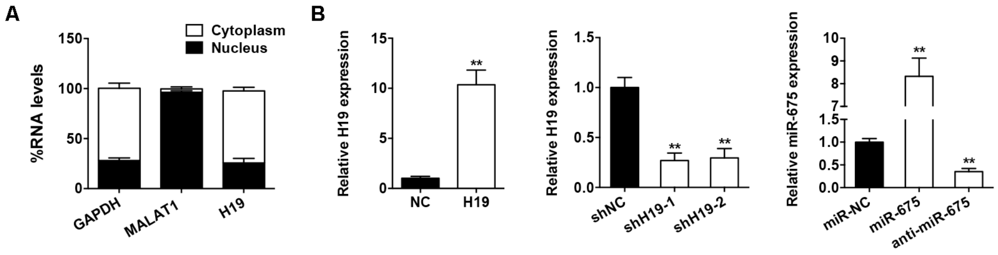

Supplementary Figure S2. Relative mRNA expression of *HDACs 4-6* in BMSCs treated with or without TSA (400 nM). Results are presented as mean ± SD.

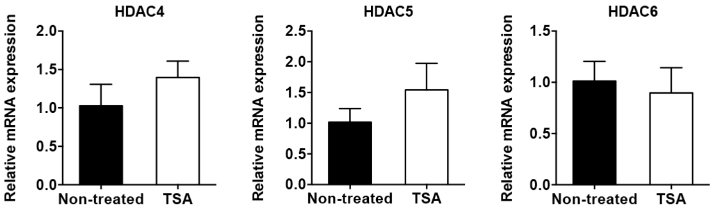

Supplementary Table S1. The miR-675 potential target sites in HDAC4 transcript according to the RNA22 software

miRNA	leftmost position of predicted targeted site	cDNA region	folding energy (in -Kcal/mol)	predicted target site	base pairs in putative heteroduplex	span of target	p-value
**has-miR-675**	**4183**	**3'UTR**	**-22.4**	**CACGGGAAGCCTTTCTGCCGCCCA**	**19**	**24**	**0.116**
has-miR-675	6166	3'UTR	-16.8	CCACTTGGGTTTTTCCACTGCAGCCA	16	26	0.0919
has-miR-675	6231	3'UTR	-17.9	AACCCAGGGACCCTTGTTGCCTG	14	23	0.186
has-miR-675	6602	3'UTR	-12.4	GGTCTTGGGACTTGTTTGACTT	14	22	0.18
has-miR-675	6671	3'UTR	-14.7	AGGAGCTGGTCCATTCCCCATTC	13	23	0.0978
has-miR-675	6766	3'UTR	-17.9	CTCTGGGGATCCTGCCACCT	16	20	0.124
has-miR-675	8170	3'UTR	-18.5	CGCCGCCCGCCCTCACTGGCCT	16	22	0.147
has-miR-675	7360	3'UTR	-23.4	CGCTGGGACTGGCCGCCCC	15	19	0.261

The bold item indicates the predicted target site of maximum likelihood

Supplementary Table S2. The miR-675 potential target sites in HDAC5 transcript according to the RNA22 software

miRNA	leftmost position of predicted targeted site	cDNA region	folding energy (in -Kcal/mol)	predicted target site	base pairs in putative heteroduplex	span of target	p-value
**has-miR-675**	**4443**	**3'UTR**	**-19.1**	**AGAACCAGGTCTGCTCCCCACCT**	**13**	**23**	**0.261**
has-miR-675	3925	3'UTR	-12.3	GTCCCTCCCCCCATTTTCCTGCATTC	13	26	0.023
has-miR-675	5208	3'UTR	-13.7	TCATGTAAACCCACTCCTTGCTT	14	23	0.221
has-miR-675	5162	3'UTR	-17.8	GGCTCCCGGCCCCTTACCCACTC	16	23	0.127
has-miR-675	4210	3'UTR	-14.7	TTTCCCCTTCTTTCGTCCTCACCA	13	24	0.00691

The bold item indicates the predicted target site of maximum likelihood

Supplementary Table S3. The miR-675 potential target sites in HDAC6 transcript according to the RNA22 software

miRNA	leftmost position of predicted targeted site	cDNA region	folding energy (in -Kcal/mol)	predicted target site	base pairs in putative heteroduplex	span of target	p-value
**has-miR-675**	**3832**	**3'UTR**	**-19.1**	**AGAATACGGTCCCTCTTCACCT**	**13**	**22**	**0.127**
has-miR-675	3903	3'UTR	-18.7	GATGAGGGGTAGCCTCCCACTGCATCC	15	27	0.0179

The bold item indicates the predicted target site of maximum likelihood

Supplementary Table S4. Sequences of RNA and DNA Oligonucleotides

Name	Sense Strand/Sense Primer (5'-3')	Antisense Strand/Antisense Primer (5'-3')
**Primers for qRT-PCR**		
H19	GCACCTTGGACATCTGGAGT	TTCTTTCCAGCCCTAGCTCA
PPARγ	GCTGTTATGGGTGAAACTCTG	ATAAGGTGGAGATGCAGGTTC
C/EBPα	GCAAGGCCAAGAAGTCGGTGGAC	TGCCCATGGCCTTGACCAAGGAG
FABP4	AGCACCATAACCTTAGATGGGG	CGTGGAAGTGACGCCTTTCA
HDAC1	CCGCATGACTCATAATTTGC	TGGAGCGCAAGAATTTAATG
HDAC2	AATTTGCGCATGTTACCTCA	TATCCGCTTGTCTGATGCTC
HDAC3	GATGGCATTGATGACCAGAG	GATCACAGCCCAGAGAGTCA
HDAC4	CGTCCCGGTACTTGTATGTG	ATTTCCACGGAAACGATAGC
HDAC5	TTCTTTGGACCAGAGTTCCC	GTTGGGTTCAGAGGCTGTTT
HDAC6	CGGAGGGTCCTTATCGTAGA	GTAGCGGTGGATGGAGAAAT
GAPDH	GGTCACCAGGGCTGCTTTTA	GGATCTCGCTCCTGGAAGATG
miR-675 RT primer	GTCGTATCCAGTGCAGGGTCCGAGGTATTCGCACTGGATACGACCACTGTG	
miR-675	GTGCTGGTGCGGAGAGG	GTGCAGGGTCCGAGGT

U6	CTCGCTTCGGCAGCACA	AACGCTTCACGAATTTGCGT
**Mimics**		
miR-675 mimic	UGGUGCGGAGAGGGCCCACAGUG	CUGUGGGCCCUCUCCGCACCAUU
mimic NC	UCACAACCUCCUAGAAAGAGUAGA	UACUCUUUCUAGGAGGUUGUGAUU
**siRNAs**		
siHDAC4	CGACAGGCCUCGUGUAUGATT	UCAUACACGAGGCCUGUCGTT
siHDAC5	GGACUUCUCUGCACAGCAUTT	AUGCUGUGCAGAGAAGUCCTT
siHDAC6	GAGGGUCCUUAUCGUAGAUTT	AUCUACGAUAAGGACCCUCTT
siNC	UUCUCCGAACGUGUCACGUTT	ACGUGACACGUUCGGAGAATT

Abbreviations: C/EBPα, CCAAT-enhancer-binding proteins-α; FABP4, fatty acid binding protein 4; GAPDH, Glyceraldehyde 3-phosphate dehydrogenase; HDAC, histone deacetylases; PPARγ, peroxisome proliferator-activated receptor-γ.
